# Effects of Workplace Supervised Exercise on Psychological Capital: An Intervention Study

**DOI:** 10.3390/sports13010002

**Published:** 2024-12-24

**Authors:** Carmen M. Salvador-Ferrer, Pedro A. Diaz-Fúnez, Álvaro Carrera-Ruiz, Montserrat Monserrat-Hernández, Enrique G. Artero, Miguel A. Mañas-Rodríguez

**Affiliations:** 1 Work and Organizational Psychology Research Team (IPTORA), University of Almería, 04120 Almería, Spain; cmsalva@ual.es (C.M.S.-F.); marodrig@ual.es (M.A.M.-R.); 2 Area of Physical Education and Sport & UAL Activa Program, University of Almería, 04120 Almería, Spain; alvarocarrera@ual.es (Á.C.-R.); artero@ual.es (E.G.A.); 3 Social and Cultural Anthropology Laboratory, University of Almería, 04120 Almería, Spain; mmh548@ual.es

**Keywords:** workplace supervised exercise (WSE) program, psychological capital (PC), gender, public administration, intervention, longitudinal study

## Abstract

Public employees face a growing array of professional challenges, and psychological capital (PC) has emerged as a valuable resource to address them. Workplace supervised exercise (WSE) programs can improve physical fitness and personal resources, though their adoption in organizations is limited. This study examines the impact of a long-term WSE program on the psychological capital of public administration employees. Fifty-five participants completed PC questionnaires and attended at least 40% of the sessions; 49.1% were women, and 40% were over 50 years old (age range: 24–73 years). A quasi-experimental design without a control group was used with a longitudinal design, and a nine-month WSE program (from October to June) was implemented. The data show gender-specific variations in PC patterns. Among men, incremental increases were observed over the three measurement intervals, with statistical significance achieved only in self-efficacy and hope. Among women, an upward trend was observed between October and February without statistical significance, followed by a slight decline. The results suggest that the WSE program may enhance personal resources, particularly hope and self-efficacy, highlighting the importance of tailoring WSE programs to the specific characteristics of the target group.

## 1. Introduction

Public employees are the backbone of any modern society, ensuring access to essential services for the population. For this reason, safeguarding their well-being should be a priority for all institutions [1]. However, factors such as work overload, high levels of bureaucracy and rigidity, and lack of social recognition can negatively impact their emotional states [2]. Finding strategies to strengthen these emotional states is crucial for helping public employees manage growing demands and improve their performance [3]. Interventions are key elements in the development of healthy organizational environments [4].

Psychological capital (PC) is a set of skills that enable individuals to navigate daily challenges. This construct comprises four key components: self-efficacy, hope, optimism, and resilience. Bandura et al. [5] define self-efficacy as an individual’s confidence in their personal abilities. Salanova et al. [6] emphasize that the perception of self-efficacy is crucial across various aspects of life, particularly in work performance. Hope is understood as the belief in one’s ability to set goals and develop effective strategies to achieve them [7]. Individuals with high levels of hope not only seek efficient strategies but also explore alternative pathways to success, facilitating higher performance [8]. Optimism, as described by Forgeard and Seligman [9], refers to the tendency to adopt positive cognitive patterns. Luthans et al. [7] suggest that optimism is vital for employees to approach situations with a pragmatic and flexible mindset, allowing them to interpret events and devise solutions effectively. Finally, resilience is the ability to bounce back from adversity without significant difficulties. Resilient individuals not only recover from setbacks but are also determined to move forward and overcome challenges [10].

Research examining the relationship between physical activity and hope is limited and is mainly focused on samples from elite athletes. A study by Curry et al. [11] with college athletes found that physical training was positively correlated with increased levels of hope, as well as better academic and athletic achievements. As these athletes developed their hope, they demonstrated a greater ability to set challenging goals and greater perseverance to achieve them. More recently, a study by Martinek et al. [12] with elementary school students identified the hope of achieving success as a key element derived from exercise. The improvement in hope significantly contributed to the increased commitment of the students. Therefore, physical training and sports practice appear to be effective strategies for visualizing success in future goals and improving commitment to these goals. However, no studies have been found yet that address this relationship in workplace contexts.

The development of an optimistic mindset is positively associated with physical activity. A longitudinal study involving more than 20,000 women concluded that those who regularly exercised exhibited higher levels of optimism [13]. Furthermore, the findings of Reche et al. [14] indicate that developing optimism is a skill that enhances persistence in achieving sports goals.

Regarding the relationship between resilience and physical activity, Fasey et al. [15] found that resilience is critical to achieving success in elite sports organizations. Hartigh et al. [16] emphasize the importance of understanding and improving athletes’ resilience as a fundamental tool for ensuring successful performance. In the sports field, this capacity has been studied primarily as a precursor to competitive success. Research has shown that resilience provides athletes with an improved ability to overcome challenges [17] and allows them to adapt more easily to changing environments [18].

One of the physical activity practices that has the greatest impact on the development of healthy organizational environments is physical exercise programs designed to improve employee physical condition [4,19]. These programs are defined as a set of specific exercises aimed at improving health-related attributes or capabilities [20]. Among the various options for implementing a physical exercise program in organizations, Marín-Farrona et al. [21] suggest that the most effective interventions are those that adopt a supervised circuit training approach, combining aerobic exercises with activities to improve muscular strength. Integrating physical conditioning programs in the workplace is a recognized need by international institutions as a fundamental organizational practice to promote employee health. A clear example of this is the recommendation of the World Health Organization (WHO) [22] and, similarly, the Sustainable Development Goals (SDGs) of the United Nations (UN), which advocate for promoting health and physical activity in the workplace [23].

In this line, the work of Joubert and De Beer [19] demonstrates that exercising in the workplace improves cohesion, collaboration, and productivity in work teams with cultural, racial, and social differences in the workplace. These authors implemented sports programs to learn about their effect on employees from different groups. The data, despite the existing limitations, indicate that there is an improvement in communication, which becomes more open, in the sense of belonging. In their work, they conclude by highlighting the need for organizations to implement sports programs to improve workplace inclusiveness, thus achieving a more equitable and collaborative culture.

Despite the benefits observed in other contexts and the recommendations issued by international institutions, the implementation of workplace supervised exercise (WSE) programs in organizations remains very limited, and there are no studies analyzing the effects of long-term interventions. Barr-Anderson et al. [24], in a meta-analysis of brief interventions that integrate physical activity into organizational routines, found that although the benefits are modest, they are consistent and represent promising avenues for research. These authors conclude that it is essential to conduct studies that include longer-duration interventions, suggesting that these strategies could produce more significant and sustainable results. In this context, a research line opens to analyze how a long-term WSE program could influence the work environment, where a more pronounced effect on PC is anticipated.

Based on a systematic review carried out by Brinkley et al. [25], despite the existing interest in promoting the practice of sport in the workplace, there are no studies that conclude on its benefits. Specifically, their review showed that the benefits are not only individual but also collective, since it improves cohesion, group performance, and work performance. However, as these authors point out, most of the studies do not offer clear evidence, since the samples were not well defined, or the sports practices were not clearly specified.

The aim of this study is not only to analyze the impact of a WSE program on PC but also to identify the elements that can influence the design of interventions to maximize their effectiveness. One factor that appears to have a significant influence on the outcomes of a WSE program is gender. Authors such as Devries and Jackoby [26] emphasize that this aspect is crucial when planning physical activity intervention activities. In a study conducted in the armed forces, Yanovich et al. [27] found that the results of physical training varied according to the recruits’ gender. Furthermore, the way organizational interventions affect personal resources has shown gender differences, particularly with respect to factors associated with psychological capital [28]. Furthermore, Younas et al. [29] found that women experience higher levels of stress, anxiety, and depression compared to men in the workplace, which can influence how physical exercise affects PC development.

A useful model for conceptualizing the influence of WSE programs on PC is the Job Demands–Resources (JD-R) theory. This theory, proposed by Bakker and Demerouti [30], has been instrumental in analyzing how positive factors influence the work environment [31]. It is based on two key elements: job demands and job resources. Job demands are aspects of work that require effort and lead to physical, cognitive, or emotional stress. On the other hand, job resources are factors that help employees cope with demands and achieve their goals, playing a critical role in their overall well-being.

Within this theory, resources are divided into two categories: job resources and personal resources. Job resources are those provided by the organizational context, such as support or tools that make tasks easier and promote employee well-being. In contrast, personal resources refer to individual characteristics that employees possess, allowing them to effectively handle job demands [32], for example, mindfulness abilities, emotion management capabilities, personal well-being, or psychological capital skills. According to this model, a supervised exercise program implemented in the workplace can be considered an organizational resource aimed at enhancing employees’ personal resources, such as PC.

The objective of this study is to assess the impact of a long-term WSE program on self-evaluation PC by public administration employees, analyzing whether this effect differs between men and women. This study examines the changes that a nine-month WSE program produces in the four dimensions of PC (self-efficacy, hope, resilience, and optimism), measured at three different points over the course of an academic year (September, February, and June). Additionally, it evaluates whether the program’s effect may vary according to the participants’ gender. Based on previous scientific evidence, the following hypotheses are proposed.

**Hypothesis 1 (H1).**  *The WSE program will significantly increase the PCs of employees in its four dimensions*.

**Hypothesis 2 (H2).**  *The effect of the WSE program on PC will increase as the duration of the intervention increases.*

**Hypothesis 3 (H3).**  *The beneficial effects of the WSE program on the dimensions of psychological capital will manifest differently depending on men’s or women’s perception of the work context.*

The proposals outlined in this study represent significant scientific advancement for several reasons. First, there is limited research exploring how WSE programs impact positive psychological development. Second, most existing studies on this relationship focus on professional athletes, physical education teachers, coaches, or students. Few studies examine the influence of physical exercise on employees. Third, the limited available research does not involve long-term supervised structured training programs [33], nor does it consider factors that may affect the final impact of the WSE programs on PC, such as gender.

## 2. Materials and Methods

### 2.1. Intervention

The intervention was carried out over 9 months using a quasi-experimental method (working with established groups to identify trends) with a longitudinal design, no control group, with measures of psychological capital (PC) taken at three different time points: T1 (October), T2 (February), and T3 (June). The WSE program consisted of a total of 60 sessions throughout the intervention period. Each session lasted 60 min, with a frequency of 2 sessions per week and 48 h of rest between them, following the recommendations of the American College of Sports Medicine [34].

The WSE program was implemented at the workplace in a designated space equipped with the necessary materials. Training sessions were conducted in small groups of 3 to 6 participants, each led in person by students from the Physical Activity and Sports Sciences degree program. These students had undergone an additional, specialized training program of 120 h tailored specifically for this purpose.

#### Session Structure

- Block 1: Movement Preparation

Participants began the session by performing 10 repetitions of self-myofascial release on the soles of the feet, back, and posterior, anterior, and medial parts of the legs [35]. This was performed with the goal of activating diffuse noxious inhibitory controls (DNICs) and increasing range of motion [36].

Subsequently, participants performed 2 sets of 8–10 repetitions of an exercise designed to manage intra-abdominal pressure [37], while avoiding the Valsalva maneuver, which can elevate blood pressure [38].

Third, they carried out three manual resistance exercises, each consisting of 2 sets of 3–5 repetitions of high-intensity contractions lasting 5 s, focusing on the most relevant muscles for the strength block or those that presented the most discomfort among participants. This type of exercise promotes an increase in spinal excitability and exercise-induced hypoalgesia [39]. Additionally, the increase in analytical corticospinal demands can contribute to improving motor cortical maps [40].

- Block 2: Muscle Power

Two sets of 6–8 repetitions were performed, focusing on generating maximum power with low or low–moderate fatigue levels [41]. These actions can induce a global post-activation potentiation effect.

- Block 3: Muscle Strength

Main exercise: On the first day of the week, a hip hinge exercise was performed, and on the second day, a triple extension exercise was performed involving the hip, knee, and ankle. Between 3 and 4 sets of 4–16 repetitions were completed, maintaining between 1 and 3 repetitions in reserve (RIR) [42]. The selection of each exercise was based on the participant’s stability in the movement and the correct execution of technique throughout all repetitions. For progression, three dimensions were considered: volume of repetitions and load, range of motion, and intention of contraction.

Secondary exercises: Three exercises were performed: one for pushing, one for pulling, and a third exercise that varied depending on the day of the week. On the first day, a triple extension exercise was performed, while on the second day, a hip hinge exercise was performed. Each exercise consisted of 3–4 sets of 8–16 repetitions, maintaining 1–6 repetitions in reserve (RIR). The primary constraint for these exercises was stability, which was addressed by performing them in asymmetric and/or unilateral positions, both in terms of support and load distribution. For progression, four dimensions were considered: volume of repetitions and load, movement plane, range of motion, and intention of contraction.

- Block 4. High-Intensity Interval Training

Participants participated in 5 to 10 min of aerobic exercises using machines, incorporating high-intensity interval training (HIIT) [43], or performed strength exercises through high-intensity interval resistance training (HIIRT) [44].

- Block 5. Cool down

To conclude each session, participants performed self-myofascial release exercises targeting the major muscles worked during the session, which aids in reducing delayed onset muscle soreness (DOMS) [45].

### 2.2. Participants

The study sample consisted of 89 voluntary sign-up employees from a public university in southern Spain. This study received approval from the Human Research Bioethics Committee of the University (UALBIO2022/037). All participants were enrolled in the University’s WSE program. The criteria for inclusion in the final analysis were the following: (a) attendance at more than 40% of the WSE sessions, which was tracked using online records managed by students from the Physical Activity and Sports Sciences degree during each session, and (b) correctly completing all questionnaires at the specified times (T1, T2, T3). Consequently, 34 participants were excluded for failing to meet either of the two previous criteria, resulting in a final sample of 55 employees, representing 62% of those who started the study. The age distribution of the final sample was as follows: under 30 years = 12.7%, 31 to 40 years = 21.8%, 41 to 50 years = 25.5%, and 51 years or older = 40.0%. In terms of gender, 51% were men, and 49% were women.

### 2.3. Measures

Psychological capital (PC) has been shown to be a critical factor in fostering positive emotional states at work [46]. This research highlights the significant impact of PC on workers’ emotional and motivational responses, as well as on organizational outcomes. Public institutions can enhance their employees’ ability to cope with job demands by improving their perception of PC, which in turn would boost both well-being and organizational performance. Achieving this transformation would position these institutions as healthy organizations [47].

Psychological capital was measured using the Spanish adaptation [48] of the Psychological Capital Questionnaire (PCQ12) developed by Luthans et al. [49]. The questionnaire measures four psychological resources: self-efficacy (3 items; for example, “I feel confident presenting information to a group of colleagues”), hope (4 items; for example, “I can think of many ways to reach my professional goals”), resilience (3 items; for example, “I can handle difficult times at work because I have already experienced overcoming challenges”), and optimism (2 items; for example, “I always look on the bright side of things related to work”). All elements are scored on a six-point frequency scale ranging from 1 (strongly disagree) to 6 (strongly agree). The alpha coefficient was 0.81.

### 2.4. Statistical Analysis

Data were analyzed using SPSS software, version 29 (SPSS Inc., Chicago, IL, USA). Since the dependent variable scores (PC) did not follow a normal distribution according to the Kolmogorov–Smirnov test (*p* < 0.05), non-parametric tests were applied [50]. The Friedman test was used to examine differences in perception of the four dimensions of psychological capital over three measurement times (T1, T2, T3) for each of the two groups (men and women). Pairwise comparisons between measurement times were conducted using the Wilcoxon test as a follow-up, with Bonferroni corrections applied. The significance level was set at *p* < 0.05, *p* < 0.01, and *p* < 0.001, respectively.

## 3. Results

Table 1 presents the mean scores and standard deviations for the four dimensions of psychological capital at each measurement point. The results are disaggregated by gender, showing separate scores for women and men to analyze potential differences in the average values of these variables according to gender.

Table 1 shows that both men and women score above the midpoint of the scale (value 3) in all dimensions of psychosocial capital from the first measurement point (T1). For women, the mean scores on the dimensions of psychological capital increase from T1 to T2 but decrease at T3. On the contrary, men show a continuous upward trend at all three measurement points, with uniform increases from T1 to T2, followed by further increases at T3. For women, the highest scores are recorded at T2 in all dimensions, while men consistently achieve their maximum scores at T3.

### Longitudinal Results

The following tables present the results of mean comparisons in the dimensions of psychological capital (self-efficacy, hope, resilience, and optimism) using non-parametric tests for each gender. Table 2 presents the results for the female group.

Regarding the female group, Table 2 shows that none of the dimensions of psychological capital exhibit statistically significant differences between the values at the different measurement times.

Table 3 presents the results for the male group.

In the male group, statistically significant differences were observed in self-efficacy scores between T1 and T3 (Q: −0.714; adjusted *p*: 0.023; effect size: 0.61). Similarly, significant differences in hope scores were found between T1 and T3 (Q: −0.743; adjusted *p*: 0.021; effect size: 0.69). Comparisons of the other dimensions at different measurement times did not reveal statistically significant differences.

**Figure 1:**
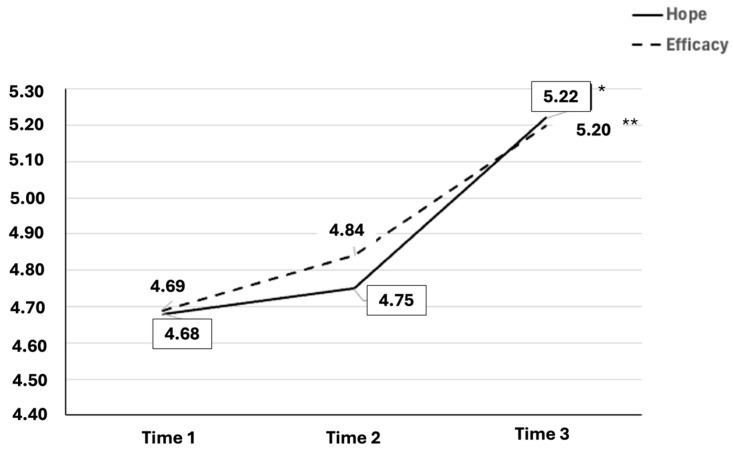
Trends in self-efficacy and hope dimensions in T1, T2, and T3 in the male group. Repeated measures ANOVA with Bonferroni test as post hoc. Hope, T1–T3 * *p* = 0.021; efficacy, T1–T3 ** *p* = 0.023.

Figure 1 illustrates the trends in self-efficacy and hope dimensions across T1, T2, and T3 for the male group.

The preceding graph depicts the significant changes in self-efficacy and hope scores within the male group. Both dimensions demonstrate a positive trajectory, particularly between T1 and T2 and more pronouncedly between T2 and T3.

## 4. Discussion

Interventions aimed at improving the physical fitness of employees have shown a positive impact on fostering healthy organizational environments [4]. However, few studies have explored the effects of long-term physical exercise programs, and the relationship between physical activity and positive outcomes for both employees and organizations remains largely unexplored. This presents a promising avenue for research, focused on examining how the implementation of a WSE program in the workplace influences the workplace environment. In this context, the assessment of psychological capital (PC) appears to play a pivotal role.

The objective of this study was to analyze the effects of a nine-month long-term WSE program on employees’ self-assessment of PC in a public administration setting, while also examining whether these effects vary by gender.

The first hypothesis posited that the WSE program would progressively increase the PC of employees in the four dimensions analyzed. This hypothesis is partially supported, as the male group exhibited a consistent increase in all dimensions of psychological capital, with statistically significant changes observed between T1 and T3 in the dimensions of self-efficacy and hope. On the contrary, the female group demonstrated a nonsignificant increase in all four dimensions of PC between measurement times 1 and 2, followed by a slight decline at T3. These findings corroborate the extensive literature supporting the positive effects of WSE programs on PC components [11,12,16,17], while also highlighting the nuance that this impact may depend on personal characteristics of employees, and it does not occur in all the dimensions of PC.

The second hypothesis proposed that the impact of the WSE program on PC would strengthen progressively with the extended duration of the intervention. This hypothesis is partially confirmed. The proposed WSE program resulted in a consistent increase throughout the intervention period for the male group. However, this pattern was not observed in the female group, where a short-term effect was evident (from T1 to T2), but this effect did not persist in T3. This finding partially supports the proposal of Barr-Anderson et al. [24], who suggested that long-term physical activity interventions would have a sustained impact on improving the workplace context.

The third hypothesis posited that the beneficial effects of the WSE program on PC dimensions will manifest differently depending on men’s or women’s perception of the work context. This hypothesis is supported by the results obtained. The influence of the WSE program on PC differs between genders. Both groups show an initial improvement from T1 to T2; however, the improvement in T3 is sustained only in the male group, while the female group shows a slight decrease in PC values between T2 and T3. This finding aligns with the proposals of Devries and Jackoby [26] and Yanovich et al. [27], who identified differences in the effects of physical training between women and men, as well as the conclusions of Cnen et al. [28] and Younas et al. [29], who reported similar disparities in PC.

### 4.1. Theoretical Implications

The preceding discussion of the results leads to the formulation of five theoretical implications of the present study. The first implication pertains to the support for the proposed positive interaction between organizational and personal resources as outlined in the Job Demands–Resources (JD-R) theory [31]. The introduction presents the JD-R theory as the conceptual framework for this manuscript, and the results corroborate the impact of the WSE program, characterized as a workplace resource, on the four dimensions of the personal resource of PC. This finding confirms the positive relationship between job resources and personal resources, thereby enhancing employees’ capacity to effectively cope with work demands [32].

The second theoretical implication is evidence that improved physical fitness among employees appears to positively influence the development of their PC. Previous research has not provided clarity on how participation in physical activity can lead to emotional, attitudinal, and behavioral changes in individuals. The intervention based on the WSE program has shown improvements in the perceptions of employees of their own abilities (self-efficacy), their expectations of goal achievement and planning strategies (hope), their belief in positive future outcomes (optimism), and their ability to cope with difficulties (resilience).

The third theoretical implication suggests that the effects of a long-term supervised exercise program on PC appear to manifest similarly within a workplace context. Most prior studies linking these two constructs have focused primarily on professional or elite athletes [11,14,15,16,17,18]. Furthermore, some research has investigated this relationship in primary school students [12]. On the contrary, this study examines the influence of a WSE program on employees within a public organization and corroborates its effects in this specific context.

The fourth theoretical implication focuses on the duration of WSE programs and their influence on outcomes. The present study indicates that the duration of the intervention program may be a crucial factor in its effectiveness, supporting the assertions made by Barr-Anderson et al. [24]. The findings reveal that the duration of the WSE programs positively affected the male group, as they experienced an increase in PC at all measurement time points; however, this improvement was limited, with significant changes occurring only in the dimensions of self-efficacy and hope. On the contrary, although the female group showed an increase in PC from T1 to T2, this enhancement did not persist from T2 to T3. These results suggest that long-term WSE programs can extend improvements in PC outcomes, but further research is needed to fully understand this process.

The final theoretical implication arises from the unequal influence of the long-term WSE program on PC based on gender, as evidenced by the results. Specifically, women and men exhibit differential responses to the intervention. In the case of women, there is a positive impact on PC values in the short term (from T1 to T2); however, this benefit dissipates by T3, returning to levels like those observed at T1. On the contrary, the male group shows a consistent improvement in PC values at all three time points, although significant changes are observed only in the dimensions of self-efficacy and hope. These findings support previous research indicating that physical exercise programs exert varying influences on individuals based on gender [25,26], which also differentiates the perception of PC [27,28].

Although these data should be treated with caution, due to the limitations of this study, the findings seem to point to the existence of important gender differences, the data being particularly striking in the case of women. In this regard, it would be useful to know what factors (cultural, social, and/or occupational) could be influencing the data obtained.

### 4.2. Practical Implications

The findings of this study yield three implications for the practical application of WSE programs within organizations aimed at improving PC. The first implication underscores the advantages associated with the implementation of WSE programs. The results indicate that such programs exert a positive effect on employees’ PC. While the data reveal differences in the magnitude of benefits based on gender, and not all dimensions of PC exhibited statistically significant improvements, the findings are nevertheless encouraging. They support the rationale for implementing these programs within businesses and institutions to improve employee well-being and performance.

The second practical implication pertains to the enhanced benefits associated with the implementation of structured long-term WSE programs within organizations. As observed in the male group, the advantages of this program on PC appear to increase with prolonged participation in physical activity. Although the benefits did not persist for the female group by T3, the program demonstrated effectiveness during the initial phase, specifically between the October and February measurements, which coincided with a four-month intervention period. This underscores the importance of sustained participation in WSE programs to maximize their positive impact on PC.

The third practical implication focuses on the differential benefits of the WSE programs for PC based on gender. This work could be seen as opening a debate on gender differences and invites reflection not only on the need to implement employee wellness improvement strategies but also on what kind of specific actions are needed to ensure the success of the outcome according to the gender of the participants. The findings of this study indicate that from February to June (T2 to T3), the positive impact of the program on PC was sustained in the male group, while it decreased in the female group. These results suggest that the design of intervention programs within organizations should account for the unique characteristics and needs of different demographic groups to improve their positive effects across all participants. By tailoring programs to address these specific differences, organizations can optimize the benefits of WSE programs for all employees.

### 4.3. Limitations and Future Research

Following the analysis of the study’s results, this research identifies some limitations that provide important considerations for future investigations. The first limitation is related to the quasi-experimental design, since the data could be biased, and the behavior of the participants could be conditioned by the research context.

The second limitations relate to the specificity of the sample. This study was conducted with a relatively small group of public sector employees in Spain. The effects of these interventions may be different in other populations with different occupational characteristics. Therefore, future research should aim to examine the effects of WSE programs on PC in different organizational contexts, such as private sector management and non-governmental organizations (NGOs). In addition, it would be beneficial to include employees of different nationalities to determine whether the effects observed in this study are consistent across settings.

The third limitation arises from the duration of the intervention. Conducting a study with an extended intervention period of nine months, combined with three measurement time points and voluntary participation, results in a limited number of participants and a high attrition rate. Furthermore, the stringent inclusion criteria led to a final sample size of 55 subjects. Future research should aim to develop strategies to mitigate the high attrition rates commonly observed in such studies.

The fourth limitation pertains to the absence of a control group for comparison with the experimental group. Given that this study involved a longitudinal program aimed at enhancing organizational competencies and improving employee resources, the possibility of establishing a reference control group was restricted. Subjects initially designated as the control group, as well as those who did not meet the inclusion criteria, failed to complete the PC measurements. Consequently, this lack of a control group has hindered the ability to compare results between the two groups. Future studies investigating the effects of organizational intervention programs based on WSE should prioritize the inclusion of a control group to accurately assess the true impact of the advancements achieved by the experimental group.

The fifth limitation pertains to the results observed within the female cohort. Previous studies have indicated that the effects of WSE programs and PC may differ depending on gender. In summary, the absence of a control group and the limited sample size may reduce the generalizability of the conclusions. However, these studies implied a different rate of improvement associated with this characteristic in relation to the intervention. The current study did not incorporate control elements that could elucidate the observed decrease in PC values among women between T2 and T3. For example, objective measures of the physical benefits achieved or the potential increase in stress levels resulting from the progression of the academic year could provide valuable information. Future research should consider incorporating such factors to better account for these differences.

## 5. Conclusions

The present study, despite certain limitations regarding sample size, environment, and methodology, highlights the potential positive effects of implementing a long-term WSE program on the PC of employees within a public administration. The data indicate that the benefits of such interventions are not immediate, as significant improvements were observed in only two of the dimensions analyzed (hope and self-efficacy) after nine months of intervention. Furthermore, the findings reveal gender-based differences in the effects of the program, highlighting the need to tailor organizational intervention programs to the specific characteristics of the groups and individuals involved. This study serves as an initial step and paves the way for future research aimed at enhancing employees’ physical condition and health. The goal is to equip them with the resources needed to effectively manage work demands, achieve their objectives, and improve their overall well-being.

## Figures and Tables

**Table 1:** Means, 95% CI, and S.D. in the dimensions of psychological capital at each measurement time for men and women.

	Women		Men	
	Mean (95%CI)	Stand. Dev.	Mean (95%CI)	Stand. Dev.
Self-efficacy T1	4.85 (4.52 to 5.18)	0.83	4.69 (4.37 to 5.00)	0.81
Self-efficacy T2	4.98 (4.70 to 5.26)	0.71	4.84 (4.52 to 5.17)	0.83
Self-efficacy T3	4.69 (4.31 to 5.06)	0.95	5.20 (4.89 to 5.50)	0.78
Hope T1	4.60 (4.30 to 4.90)	0.75	4.68 (4.39 to 4.97)	0.74
Hope T2	4.74 (4.49 to 4.98)	0.62	4.75 (4.41 to 5.10)	0.89
Hope T3	4.58 (4.24 to 4.92)	0.85	5.22 (4.96 to 5.48)	0.67
Resilience T1	4.37 (3.97 to 4.76)	1.00	4.72 (4.40 to 5.04)	0.81
Resilience T2	4.74 (4.45 to 5.02)	0.72	4.83 (4.48 to 5.18)	0.89
Resilience T3	4.61 (4.25 to 4.97)	0.90	5.14 (4.81 to 5.46)	0.84
Optimism T1	4.48 (4.06 to 4.89)	1.04	4.78 (4.44 to 5.12)	0.88
Optimism T2	4.83 (4.46 to 5.19)	0.91	4.85 (4.41 to 5.29)	1.12
Optimism T3	4.51 (4.10 to 4.93)	1.05	5.16 (4.80 to 5.51)	0.91

Note: T1 (Time 1), T2 (Time 2), T3 (Time 3).

**Table 2:** Comparison of measurement times for the dimensions of psychological capital in women.

	Statistic	Stand. Error	Adjust. Sig.
Self-efficacy T1-Self-efficacy T2	−0.241	0.272	1.000
Self-efficacy T2-Self-efficacy T3	−0.426	0.272	0.353
Self-efficacy T1-Self-efficacy T3	−0.185	0.272	1.000
Hope T1-Hope T2	−0.111	0.272	1.000
Hope T2-Hope T3	−0.278	0.272	0.922
Hope T1-Hope T3	−0.167	0.272	1.000
Resilience T1-Resilience T2	−0.481	0.272	0.231
Resilience T2-Resilience T3	−0.074	0.272	1.000
Resilience T1-Resilience T3	−0.407	0.272	0.403
Optimism T1-Optimism T2	−0.278	0.272	0.922
Optimism T2-Optimism T3	−0.111	0.272	1.000
Optimism T1-Optimism T3	−0.167	0.272	1.000

Note: T1 (Time 1), T2 (Time 2), T3 (Time 3).

**Table 3:** Comparison of measurement times for the dimensions of psychological capital in men.

	Statistic	Stand. Error	Adjust. Sig.	Effect Size
Self-efficacy T1-Self-efficacy T2	−0.357	0.267	0.544	
Self-efficacy T2-Self-efficacy T3	−0.357	0.267	0.544	
Self-efficacy T1-Self-efficacy T3	−0.714	0.267	0.023	0.61
Hope T1-Hope T2	−0.196	0.267	1.000	
Hope T2-Hope T3	−0.518	0.267	0.158	
Hope T1-Hope T3	−0.743	0.267	0.021	0.69
Resilience T1-Resilience T2	−0.071	0.267	1.000	
Resilience T2-Resilience T3	−0.446	0.267	0.285	
Resilience T1-Resilience T3	−0.518	0.267	0.158	
Optimism T1-Optimism T2	−0.107	0.267	1.000	
Optimism T2-Optimism T3	−0.321	0.267	0.687	
Optimism T1-Optimism T3	−0.429	0.267	0.326	

Note: T1 (Time 1), T2 (Time 2), T3 (Time 3).

## Data Availability

Data availability statements are available in the University of Almeria Institutional Repository at https://repositorio.ual.es/handle/10835/4505 (accessed on 8 December 2024).
